# Mélanome anorectal primitif

**DOI:** 10.11604/pamj.2015.21.65.6838

**Published:** 2015-05-28

**Authors:** Mohamed Moukhlissi, Hasna Derouich, Soufia Majdoul, Sanaa Naoumi, Nesserine Bennani, Mehdi Karkouri, Fouad Haddad, Wafaa Badre, Abdellatif Benider

**Affiliations:** 1 Departement d'Oncologie, CHU Ibn Rochd, Casablanca, Maroc; 2 Departement de Gastro-Entérologie, CHU Ibn Rochd, Casablanca, Maroc; 3 Departement d'Anatomopathologie, CHU Ibn Rochd, Casablanca, Maroc

**Keywords:** Mélanome anorectal, diagnostic, traitement, pronostic, anorectal melanoma, diagnosis, treatment, prognosis

## Abstract

Nous proposons une étude rétrospective réalisée au sein du département de gastro-entérologie et d'Oncologie du CHU Casablanca colligeant tous les cas des mélanomes ano-rectaux primitifs, sur une période de 15 ans (du 1997 au 2012). Notre série comportait 14 patients, 8 hommes et 6 femmes avec une moyenne d’âge de 60,5 ans. Les signes cliniques étaient dominés par les rectorragies et le syndrome rectal (plus de 80% des malades). L'aspect tumoral noirâtre à été noté chez la moitié des malades. L'examen endoscopique a révélé une prédominance des lésions ulcéro-bourgeonnantes. Dans 5 cas la tumeur était plus haut située entre 5 à 8 cm de la marge anale. Le bilan d'extension avait décelé des métastases ganglionnaires, osseuses ou viscérales chez 7 malades. Un traitement chirurgical a été pratiqué chez 50% des malades (7 cas). Il a consisté en une exérèse locale isolée (2 cas) ou associée à une radiothérapie (2 cas) et une amputation abdomino-périnéale dans 3 cas. Quatre malades ont reçu une chimiothérapie et/ou radiothérapie palliative et dans deux cas on s'est contenté d'un traitement symptomatique. L’évolution a été marquée par une récidive chez les 2 patients traités par exérèse locale, dont un a été bénéficié d'une amputation abdomino-pelvienne de rattrapage et un des trois patients traités par chirurgie radicale. Deux patients sont en rémission complète après 36 mois de recul.

## Introduction

Les mélanomes malins sont des tumeurs malignes qui se développent à partir du système pigmentaire. La localisation anorectale se caractérise par sa rareté, l'absence de standards quant à la prise en charge et par son pronostic sombre. Nous rapportons une série de cas de mélanome ano-rectal primitif.

## Méthodes

Nous avons mené une étude rétrospective des cas de mélanomes ano-rectaux primitifs pris en charge au service de gastro-entérologie et d'oncologie sur une période de 15 ans du janvier 1997 - Décembre 2012. Pour réaliser cette étude on s'est basé sur l’étude des dossiers médicaux des patients et l'analyses des registres des examens proctologiques réalisés durant cette période. Quatorze cas ont été observés. La confirmation du diagnostic a été faite chez tous les malades par une étude histologique des biopsies réalisées lors de la rectoscopie, et après un examen cutané minutieux et ophtalmologique pour éliminer une autre origine du mélanome. Un bilan d'extension a été réalisé par la suit permettant de classer ces malades en trois stades selon la classification de Slingluff obligatoire pour la suite de la prise en charge notamment thérapeutique.

## Résultats

**Figure 1:**
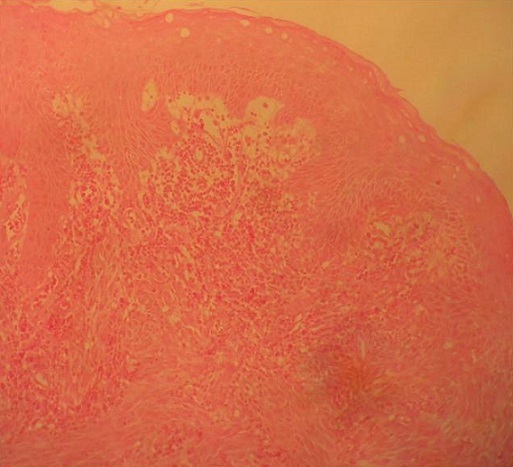
Muqueuse de type malpihien (anale). Dans le chorion, on note une prolifération tumorale à cellules fusiformes agencées en faisceaux et à cellules épithéloides globuleuses agencées en thèques

**Figure 2:**
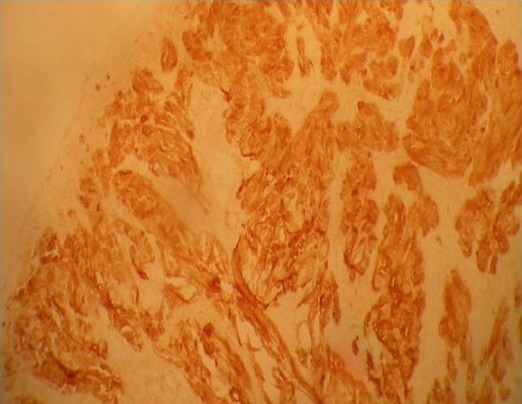
Les cellules tumorales expriment le PS100

**Figure 3:**
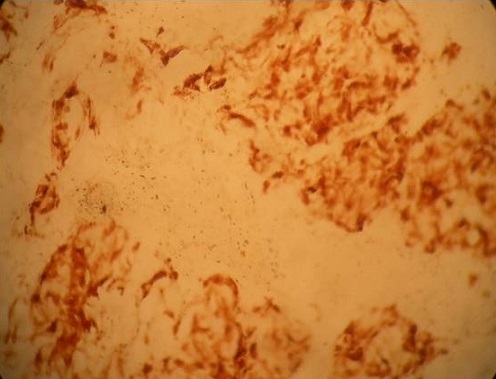
Les cellules tumorales expriment le HMB45

La moyenne d’âge était de 60,5 ans (45 à 83 ans). Il s'agissait de 8 hommes et 6 femmes. Des antécédents d'hémorroïdectomie ont été trouvés dans 3 cas. Les signes cliniques étaient dominés par les rectorragies et le syndrome rectal (Tableau 1). L'aspect tumoral noirâtre à été noté chez la moitié des malades. L'examen endoscopique a révélé une prédominance des lésions ulcéro-bourgeonnantes chez 11 malades, et dans 3 cas on a noté des lésions polypoïdes. Dans 5 cas la tumeur était plus haut située entre 5 à 8 cm de la marge anale. L’étude histologique des biopsies a conclu à un mélanome anorectal par la présence d'une prolifération tumorale à cellules fusiformes agencées en faisceaux et à cellules épithéloides globuleuses agencées en thèques (Figure 1) dans tous les cas avec un aspect achromique dans 2 cas complétée par une étude immunohistochimique confirmant le mélanome en montrant une réaction positive à la protéine S 100 (Figure 2) et l'HMB 45 (Figure 3).

**Tableau 1:** La symptomatologie à la consultation des patients

Symptomatologie	Nombre de malades
Rectorragies	10
Syndrome rectal	6
Amaigrissement	6
Prurit	2
Diarrhées	1
Syndrome anémique	1

**Figure 4:**
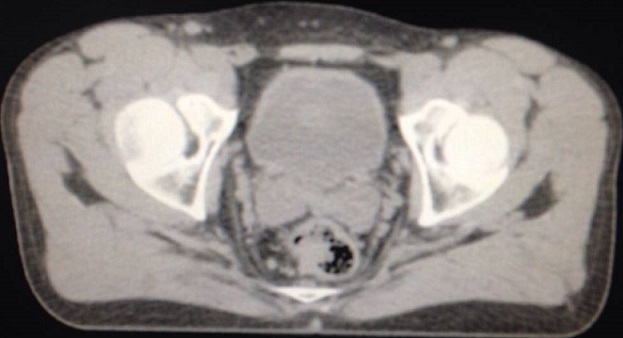
Image scannographique montrant un processus tumoral bourgeonnant non sténosant, avec infiltration du méso rectum. La biopsie de la masse a conclu un mélanome malin

Un bilan d'extension a été réalisé, Il comportait un examen clinique complet et minutieux. Le bilan paraclinique comportait une tomodensitométrie thoraco-abdomino-pelvienne à la recherche d'une extension locorégionale et à distance (Figure 4), une colonoscopie à la recherche d'autres lésion colique. Le Pet Scanner n'a été réalisé que chez deux malades. La classification de Slingluff nous a permis de classer nos malades en trois stades, localisés chez 7 malades (50% des cas), stade II par atteinte ganglionnaire chez 4 malades, une extension métastatique osseuse et/ou hépatique (stade III) a été observée chez 3 malades.

Un traitement chirurgical a été pratiqué chez 50% des malades (7 cas). Il a consisté en une exérèse locale isolée (2 cas) ou associée à une radiothérapie (2 cas) et une amputation abdomino-périnéale dans 3 cas. Quatre malades ont reçu une chimiothérapie et/ou radiothérapie palliative et dans deux cas on s'est contenté d'un traitement symptomatique.

L’évolution a été marquée par une récidive chez les 2 patients traités par exérèse locale, dont un a été bénéficié d'une amputation abdomino-pelvienne de rattrapage et un des trois patients traités par chirurgie radicale. Deux patients sont en rémission complète après 36 mois de recul.

## Discussion

Le mélanome malin ano rectal (MMAR) est une affection rare (1 à 3% de tous les mélanomes) et moins de 1% des cancers ano-rectaux [1–3]. Il survient à tous les âges avec un pic de fréquence vers la 6ème décade. Il existe une légère prépondérance féminine, sa faible prévalence explique l'absence des protocoles et des schémas thérapeutiques validés.

Sur le plan étiopathogénique, l'hypothèse d'une irritation chronique reste la plus probable, vue l'exclusion de l'exposition solaire dans cette localisation, quant au diagnostic, il ne peut être retenue que devant l'absence de toute localisation synchrone (peau, oeil), et l'absence d'antécédents d'exérèses de mélanome, quelque soit sa localisation, ce qui était le cas chez tous nos malades. La localisation rectale du mélanome est le plus souvent secondaire à une infiltration de la muqueuse rectale par un processus un point de départ anal à partir à partir des mélanocytes normalement présents dans l’épithélium malpighien de la zone pectinée et dans l’épithélium transitionnel au-dessus de la ligne pectinée [4–6].

La symptomatologie clinique est variée et non spécifique, dans notre étude elle est surtout dominée par des rectorragies et syndromes rectaux. L'examen clinique peut noter d'emblé une masse prolabée par l'anus ou une coloration noirâtre. À l'examen proctologique, le mélanome anorectal primitif se présente le plus souvent comme une tumeur ulcéro-végétante ou une lésion polypoïde et pédiculée. La couleur noirâtre caractéristique du mélanome est présente dans notre série chez le tiers des malades.

La confirmation histologique du diagnostic mélanome ano-rectal est identique à celui des mélanomes d'autres localisations et est obligatoire pour le retenir et ce par la mise en évidence du pigment mélanique sein de la tumeur par la coloration classique de FONTANA [6–9]. Les mélanomes malins sont connus pour leur diversité de caractéristiques histologiques et imitent un certain nombre de tumeurs [10], le recours à l'immunohistochimie peut s'avérer nécessaire dans les formes atypiques en particulier les formes achromiques, on utilisant utilisant l'anticorps anti-protéine S100 et l'anticorps anti- HMB45, plus spécifique des mélanomes parfois l'anticorps ant-vimentine peut elle aussi être utilisé comme marquer des mélanocytes, dans notre étude le recours à l'immunohistochimie à été fait dans 4 cas. Le mélanome est une maladie polymétastatique par la dissémination lymphatique et surtout hématogène.

En l'absence des consensus clairs par manque d’études randomisées, les moyens thérapeutiques sont représentés essentiellement par la chirurgie réalisant le plus souvent une amputation abdomino-périnéale (AAP) avec curage ganglionnaire inguinal et pelvien, qui semble être un facteur pronostique majeur influençant la survie [11, 12]. Ou une exérèse localisée de la tumeur, le recours à l'une des méthodes reste toujours un sujet de controverse. Cependant, des études récentes montrent que une exérèse tumorale épargnant le sphincter associée à une radiothérapie à faible dose semble atteindre un contrôle local similaire à une résection abdomino-pelvienne [11, 13, 14]. Trois de nos malades ont subis une AAP et deux patients une résection transanale.

D'autres moyens thérapeutiques comme la chimiothérapie et la radiothérapie sont utilisées jusqu’à l'heure actuelle comme traitement palliatif car leur efficacités dans la prise en charge curatif du mélanome reste à approuver. Des études convergent actuellement vers l'immunothérapie qui elle aussi n'a pas fait son preuve en matière d'efficacité [15, 16]. Dans notre série le recours à des traitements palliatifs par la chimiothérapie et/ou la radiothérapie à été noté dans 50% des cas.

## Conclusion

Malgré le progrès que connait le domaine de la cancérologie, le pronostic des mélanomes anorectaux reste redoutable du fait du retard diagnostique et du potentiel malin élevé. Le traitement de base à l'heure actuelle est dominé par la chirurgie.
